# Levels of attention and task difficulty in the modulation of interval duration mismatch negativity

**DOI:** 10.3389/fpsyg.2015.01619

**Published:** 2015-10-27

**Authors:** Alana M. Campbell, Deana B. Davalos

**Affiliations:** ^1^Department of Psychiatry and the UNC Carolina Institute for Developmental Disabilities, University of North Carolina at Chapel Hill, Chapel Hill, NC, USA; ^2^Department of Psychology, Colorado State University, Fort Collins, CO, USA

**Keywords:** temporal processing, time perception, dMMN, mismatch negativity, temporal perception, iMMN

## Abstract

Time perception has been described as a fundamental skill needed to engage in a number of higher level cognitive processes essential to successfully navigate everyday life (e.g., planning, sequencing, etc.) Temporal processing is often thought of as a basic neural process that impacts a variety of other cognitive processes. Others, however, have argued that timing in the brain can be affected by a number of variables such as attention and motivation. In an effort to better understand timing in the brain at a basic level with minimal attentional demands, researchers have often employed use of the mismatch negativity (MMN). MMN, specifically duration MMN (dMMN) and interval MMN (iMMN) have been popular methods for studying temporal processing in populations for which attention or motivation may be an issue (e.g., clinical populations, early developmental studies). There are, however, select studies which suggest that attention may in fact modify both temporal processing in general and the MMN event-related potential. It is unclear the degree to which attention affects MMN or whether the effects differ depending on the complexity or difficulty of the MMN paradigm. The iMMN indexes temporal processing and is elicited by introducing a deviant interval duration amid a series of standards. A greater degree of difference in the deviant from the standard elicits a heightened iMMN. Unlike past studies, in which attention was intentionally directed toward a closed-captioned move, the current study had participants partake in tasks involving varying degrees of attention (passive, low, and high) with varying degrees of deviants (small, medium, and large) to better understand the role of attention on the iMMN and to assess whether level of attention paired with changes in task difficulty differentially influence the iMMN electrophysiological responses. Data from 19 subjects were recorded in an iMMN paradigm. The amplitude of the iMMN waveform showed an increase with attention, particularly for intervals that were the most distinct from a standard interval (*p* < 0.02). Results suggest that the role of attention on the iMMN is complex. Both the degree of attention paid as well as the level of difficulty of the MMN task likely influence the neuronal response within a timing network. These results suggest that electrophysiological perception of time is modified by attention and that the design of the iMMN study is critical to minimize the possible confounding effects of attention. In addition, the implications of these results for future studies assessing interval duration-based MMN in clinical populations is also addressed.

## Introduction

We constantly rely on time: from information processing to executing action plans. But, is there a difference between the ability to perceive time and to use it? Clock models of time suggest that there are basic biological components of time. When one perceives interval durations, the beginning and end of the interval are marked neurologically and these marks are cognitively compared or computed to be used in perception, information processing, or higher order cognitive processes (Gibbon et al., 1997; Matell and Meck, 2000). Two recent questions in this model pertain to the role of attention to and to the duration of the interval to be timed. Much of the research in the past has focused on the importance of distinct neural substrates based on interval duration. Specifically, these substrates reflect at least two distinct processes, dependent upon the length of the duration. Timing related to sub-second intervals is referred to as time perception and conceived of as a more basic and automatic process which can be studied on both a behavioral and a physiological level (Ivry and Spencer, 2004). The sub-second processes have been argued to be more automatic and potentially more motor in nature (Lewis and Miall, 2003). In contrast, supra-second interval timing is thought to require more cognitive engagement and attention, in addition to recruiting different brain circuitry than sub-second intervals (Lewis and Miall, 2003; Wiener et al., 2010a). However, recent work has suggested that sub-second interval timing can be modulated by and interact with cognition.

Recently, Coull and Nobre (2008) have suggested that observed differences between sub- and supra-second intervals may not be completely explained by differences in the duration of the interval itself, but rather how that interval information is to be used. Explicit timing generally refers to tasks during which participants attend to the *duration* of a stimulus, specifically. Implicit timing, on the other hand, generally requires subjects to engage in tasks in which timing is a key component, but not the primary focus (Wiener et al., 2010b). Implicit timing is crucial to develop predictive patterns. Coull and Nobre describe two types of implicit timing; exogenous versus endogenous implicit timing that differ only in the awareness of, or cue toward the predictive pattern. Exogenous timing occurs passively, without a cue toward or awareness of temporal patterns in a task, while exogenous timing cues attention to temporal features within a task.

One way to increase awareness of temporal information is to allocate attention to that information. Attention has been a key cognitive mechanism of interest in terms of differentiating among the various measures of time. Research to date suggests that attention plays a large role in overall perception of time. Studies suggest that attending to the duration of a stimulus rather than another feature leads to greater accuracy in estimating the stimulus duration (Corbetta et al., 1993; Coull et al., 2004). Specifically, Coull et al. (2004) varied the degree to which attention was paid to the duration of a stimulus rather than its hue, they found that increased attention to the time increased accuracy in a behavioral response and was associated with greater brain activation in several regions of a corticostriatal network, including the pre-supplementary motor area, right frontal operculum and right dorsolateral prefrontal cortex. The current study investigates the role of attention via exogenous implicit timing tasks on electrophysiological indices of temporal perception. Specifically, the role of attention (via task goals) was assessed electrophysiologically to determine whether time processing as part of a task goal affected early passive pre-attentive components or only those that occur later and have been associated with attentional processes in past studies. Event related potentials (ERPs) have been utilized in the past to highlight differences in how the brain processes temporal information. Of the previous electrophysiological work on time processing, the Contingent Negative Variation (CNV) and P3 components have been used to assess the neural response to temporal information when one is actively attending to and involved in a timing task (Macar et al., 1999; Macar and Vidal, 2003; Pfeuty et al., 2005; Gibbons and Stahl, 2008). Unlike traditional behavioral measures of time processing, select ERPs can also measure the brain response to stimuli during tasks that vary in attentional demands. In particular, the MMN is used in paradigms requiring attention, but has also been observed in the absence of attention, as in sleeping infants (Martynova et al., 2003) and comatose patients (Tzovara et al., 2015). Thus, to investigate the roles of attention on time processing we can employ the mismatch negativity (MMN) event-related potential to provide information. The MMN is a component elicited in response to a deviant stimulus embedded in series of standard stimuli. The MMN is a difference wave computed by subtracting the average waveform in response to a standard stimulus from the averaged waveform in response to a deviant stimulus (Näätänen et al., 2007). The component is thought to reflect sensory echoic memory and is believed to be involved in determining whether changes in stimuli in the environment are different enough to warrant guiding attention to the stimuli (Näätänen et al., 2007). In clinical studies, MMN has been shown to have ecological validity in terms of predicting performance on select measures of memory and as a predictor of multiple measures of functional status (e.g., social, psychological and occupational; Kiang et al., 2007; Atkinson et al., 2012; Light and Braff, 2005).

In the case of studying time-based information, MMN is elicited by either a deviant inter-stimulus interval duration or a deviant stimulus duration. For testing the role of attention in time processing, MMN is ideal as it can be used to measure neural responses to changes in temporal information without attention to the task (Davalos et al., 2003). The MMN also allows one to assess variations in brain response based on the magnitude of changes in temporal information, with intervals that are more distinct from the standard eliciting greater neural responses (Kisley et al., 2004). Therefore we can use the MMN to compare the neural responses to time in an endogenous implicit task to an exogenous one. In this case, an unattended or passive MMN would reflect activity related to an exogenous implicit task—that is, there is no task goal specifically related to time. In contrast, an akin endogenous condition would require attention to the timed intervals and an awareness and expectation of those intervals in the task. The goal of the current study was to assess the interaction between degrees of attention as it varied based on task demand (from exogenous to endogenous) and duration of timed interval to test the cognitive temporal interaction in time processing.

There are previous studies suggesting that attention can modulate accuracy and brain activity to temporal estimation (Coull et al., 2004). Additionally, in select studies, attention has been shown to modulate the MMN for auditory stimuli (Sussman and Winkler, 2001; Grimm et al., 2004; Grimm and Schröger, 2005; Muller-Gass et al., 2006). These studies report enhanced MMN to attended deviants for both frequency and duration auditory deviants (in which the stimulus was presented for a longer or shorter period of time). While these results suggest that attention can modulate the MMN for tones of varying durations or of complex temporal psychical information, this modulation could be due to both auditory as well as temporal features. To isolate temporal features, an alternate technique could be to use the same physical tone to denote the beginning and end of an interval to be timed and modify the duration between tones. This technique would allow manipulation of timing information and could be used to assess the interaction of timing with attention. The current study sought to test if attention can also modulate the MMN elicited in response to purely sub-second temporal information. If so, this would (a) provide evidence that sub-second intervals do interact with cognitive processes and (b) provide support to the theory that timed information is processed separately from, but in a manner similar to, sensory information. Further, by varying the magnitude of the temporal deviants we can test the influence of attention on electrophysiological indices of temporal processing. It has been documented that the MMN is larger for deviants that are more distinct from the standard (Kisley et al., 2004; Näätänen et al., 2007), yet this interaction has not been tested for temporal processing. We predicted that endogenous attention tasks would elicit an interval MMN (iMMN) with greater amplitude than a passive, exogenous one. We tested increased degrees of attention demand over three increased deviant interval durations. We predicted that, consistent with prior research, deviant intervals that are more distinct from the standard would elicit a greater amplitude across ERP components. In addition, the greater degree of attention necessary for the endogenous tasks, would elicit greater amplitudes across ERP components. To test the effect of attention, we also investigated the negative component (N2) and the P3 in accordance with earlier work. Research suggests the N2-P3 complex, resulting from the deviant stimuli, vary with attention (Sussman and Winkler, 2001), particularly the P3 which has been well studied as an index of cognitive control and attention and varies with the rarity, probability and level of attention (Polich, 2007). While the second hypothesis is more clearly supported in the literature for the N2-P3 complex, the degree of effect of increased attention on the MMN amplitude is not as well supported. For that reason, it is hypothesized that the effects of an endogenous task versus an exogenous task on the MMN component will be less pronounced than the N2 and P3 components.

## Materials and Methods

### Participants

Participants were 19 undergraduate students who were recruited from the University’s psychology research pool and volunteered in exchange for receiving partial course credit for their participation in the study. Participants were screened for abnormal hearing, ever having a traumatic brain injury with loss of consciousness, current or past history of a neurological condition or psychiatric condition. All exclusion information was obtained using a demographic questionnaire. This study was carried out in accordance with the requirements of 45 CFR 46 and the Institutional Review Board at Colorado State University. All participants signed informed consent and completed the study; however, four were excluded from the passive condition due to artifacts. Full analysis was completed on the remaining 15 participants. The mean age was 19.59 years, standard deviation was 1.65, nine female.

### Electrophysiological Recordings

Electrophysiological recordings were acquired using a SynAmps2 system and Scan 4.1 software (Compumedics Neuroscan, Charlotte, NC, USA). Ag/AgCl electrodes hand placed at scalp locations Fz, Cz, Pz, and referenced through the left mastoid (off-line the average of both the right and left mastoids were used as the reference). Forehead served as ground. Measurements were taken in according to the 10–20 international placement guide (Jasper, 1958). The recordings were sampled at a rate of 1000 Hz with a 0.1 Hz high-pass and 200 Hz low-pass recording filter. Ocular movements were determined through superior and lateral eye electrodes. Impedances were below 10 kΩ.

### Procedure

Participants completed three interval MMN tasks at three levels of attention (passive, during which attention was diverted away from the MMN task in addition to low attentional load and high attentional load) while EEG data were recorded. Participants were seated comfortably with speakers placed 85 cm binaurally. In all tasks, a 50 ms 1000 Hz pure tone with a 5 ms rise and fall played at 75 dB HL marked the separation of the intervals, with a 400 ms standard inter-stimulus interval (as in our previous work, e.g., Davalos et al., 2003). As noted in our previous studies, interval durations are used to minimize the effects of non-temporal information on judgments as previous research has suggested that time based judgements can be affected by non-temporal information (e.g., sounds or words; Poynter and Homa, 1983). Deviant intervals were 310, 355 or 370 ms in duration, selected based on previous research suggesting that interval duration differences between approximately 10 and 20% are challenging, yet appropriate for assessing variability in performance in healthy controls (Davalos et al., 2003, 2005). Deviant interval durations were presented at an occurrence rate of 6.67% and were presented in counterbalanced blocks (only one deviant type per block). Forty-five deviant intervals of each type were presented amidst 630 standard intervals per block. In the passive condition, participants were told to watch a silent, closed-captioned video and ignore the tones (Davalos et al., 2003). Deviant interval durations were blocked such that only one deviant interval type occurred per block. In the low attention load condition, the recording block was separated into five blocks and participants had to report via a yes or no keypress response on a keyboard the existence of deviant intervals within the block that was presented, thus requiring them to pay attention to the intervals between the tones. The high attention condition mimicked the low, except it required participants to keep track of and report via keypress the number of the deviant intervals in each block, requiring a greater degree of engagement in the task (Schwartze et al., 2011). While we cannot rule out that working memory may have also been employed during the high attention task, we selected the task as counting auditory stimuli has often been used to assess both sustained attention and selective attention in past studies (Dinkelbach et al., 2015). For the attention conditions, participants were presented with blocks in which no deviant interval occurred. In these blocks, 45 standards were selected to create the ERP in response to the standard interval. Both the order of the levels within the tasks as well as the task order were counterbalanced. Task blocks were counterbalanced using a pseudo Latin square design.

### EEG Data Analysis

**FIGURE 1:**
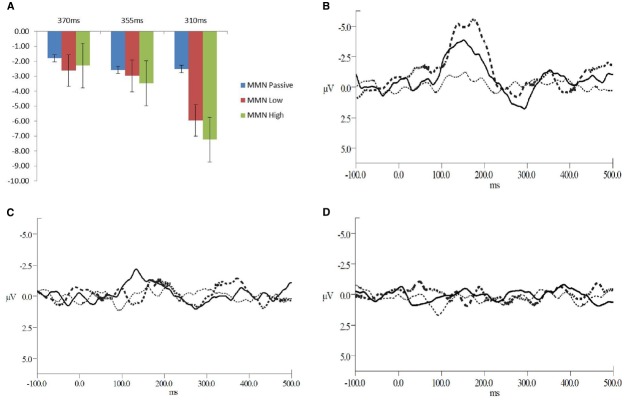
**(A)** Mean amplitudes for the MMN peaks across the deviant interval durations and levels of attention **(B–D)** Grand average waveforms for all levels of attention at **(B)** the 310 ms deviant interval duration, **(C)** 355 ms deviant interval duration, and **(D)** the 370 ms deviant interval duration. The solid line represents the low level of attention, the dashed line for the high level of attention and the dotted line for the passive attention condition.

Recordings were epoched from –100 to 500 ms post stimulus onset. Trials exceeding ±100 *μ*Vs and trials containing blinks were excluded from further analysis. A minimum of 60% of the trials remained after artifact rejection. The remaining epochs were baseline corrected, averaged and filtered between 0.1 and 30 Hz with a 0 phase shift filter and 24 dB/octave rolloff, for both standard and deviant interval trials. For each participant the average waveform in response to the standard (all for the passive condition and exemplars from each block for the low and high attention conditions) and deviant intervals were calculated. The N2 was defined as the negative-most peak occurring between 120 and 250 ms post stimulus onset at electrode Fz. The P3b peak was defined as the greatest positive peak within 250 and 500 ms post-stimulus onset at electrode Pz. The MMN reflected the greatest negative peak occurring between 120 and 300 ms post stimulus observed in electrode Fz in the difference waveform obtained by subtracting the standard from deviant interval waveforms. The MMN component is most prominent in Fz. The grand-average low and high attended waveforms compared to passive are presented in Figure 1. The mean individual amplitudes are reported in Table 1.

**TABLE 1:** **Amplitude of the MMN, N2, and P3 peaks**.

**Component**		**MMN**			**N2**			**P3**		
**Condition**		**Passive**	**Low**	**High**	**Passive**	**Low**	**High**	**Passive**	**Low**	**High**
	370 ms	–1.81	–2.28	–2.62	–1.64	–1.83	–2.45	3.24	3.92	3.17
Deviant Interval	355 ms	–2.58	–3.47	–2.98	–2.15	–2.46	–2.33	2.76	4.04	3.24
	310 ms	–2.51	–7.25	–5.95	–1.79	–5.46	–4.42	2.84	4.22	3.47

The amplitudes of the MMN for passive, low, and high attention from the MMN difference wave at electrode Fz. The N2 and P3 peaks from the deviant interval duration waveforms across conditions.

### Statistical Analyses

A 3(Attention: passive, low, high) × 3(deviance difficulty: 310, 355, 370 ms) repeated measures analysis of variance (ANOVA) assessed the interaction and main effects of attention and deviance duration. In the case of violated sphericity, the Huynh-Feldt correction was used.

## Results

### EEG: Attention and Interval Duration Influence on the MMN

A repeated measures ANOVA revealed a main effect of attention [*F*(2,22) = 4.03, *p* = 0.05, $\eta_{p}^{2}$ = 0.27]. Corrected follow-up comparisons showed that both high and low levels of attention elicited larger MMNs than passive (*t* = 3.84, *p* = 0.003 and *t* = 2.24, *p* = 0.046). However, the high and low attention conditions did not differ from each other (*t* = 0.58, *p* = 0.57). There was also a main effect of interval duration [*F*(2,22) = 9.56, *p* = 0.001, $\eta_{p}^{2}$ = 0.47]. The deviant 310 ms interval elicited a larger MMN than the 355 ms (*t* = 2.82, *p* = 0.02) and the deviant 370 ms interval (*t* = 3.71, *p* = 0.003). Importantly, there was an interaction of attention with interval duration [*F*(4,44) = 3.22, *p* = 0.02, $\eta_{p}^{2}$ = 0.23]. The deviant 310 ms intervals showed larger responses than 355 ms (*t* = 3.33, *p* = 0.007) or 370 ms (*t* = 3.48, *p* = 0.005) for low attention. The deviant 310 ms interval also induced larger responses than 355 ms (*t* = 2.21, *p* = 0.05) or 370 ms (*t* = 3.70, *p* = 0.004) deviant intervals in the high attention condition. Within the passive attention level, no differences emerged between interval deviant durations.

### EEG: Attention and Interval Duration Influence on the N2P3

Repeated measures ANOVAs were conducted on the peaks extracted from the waves in response to the deviant intervals for the N2 and P3. For the N2, there was a main effect for interval duration [*F*(2,22) = 4.08, *p* = 0.03, $\eta_{p}^{2}$ = 0.27] with the 310 ms deviant duration eliciting a wave with a greater amplitude than the 370 ms deviant (*t* = 2.33, *p* = 0.04). There was also an attention by interval duration interaction for the N2 [*F*(4,44) = 3.37, *p* = 0.02, $\eta_{p}^{2}$ = 0.23]. Follow-up comparisons revealed the N2 to have the greatest amplitude in response to the 310 ms deviant, particularly in the passive condition compared to the low attention (*t* = 2.43, *p* = 0.03) and to a lesser degree the high attention condition (*t* = 2.08, *p* = 0.06). The P3 analysis revealed a marginal effect of attention [*F*(2,22) = 3.24, *p* = 0.059, $\eta_{p}^{2}$ = 0.23]. No other main effects or interactions were found to be significant for the P3.

### Behavioral Results

We recorded behavioral data as a manipulation of attention level, but behavioral performance was not the primary focus of the study. Nevertheless, a relationship was observed between level of difficulty of the task and accuracy in detecting deviants. In the low attention condition participants correctly identified 49.12% of the duration deviant intervals. In the high attention condition, on only 19.37% of the trials were participants able to report a count of the number of deviants detected. The chi-square tests comparing behavioral performance across conditions was 14.05 (*p* = 0.0001).

## Discussion

The roles of task difficulty and attention have long been examined in the temporal processing literature. In the current study we report an interaction between level of attention and task difficulty. While previous studies have focused on the role of sub-second temporal perception versus supra-second temporal estimation as a means of better understanding overall time processing, recent studies have suggested that there may be a different factor that warrants consideration. Specifically, Coull and Nobre discuss the importance of knowing how that interval information is to be used (Coull and Nobre, 2008). And while the change in the MMN has been noted to auditory stimuli of varying durations (see Näätänen et al., 2007 or Sussman, 2007 for reviews) the interaction between level of attention and the iMMN has not been examined.

In the current study, we sought to examine the role of task and attention in implicit timing by varying the degree of attention to temporal duration information that differed in level of difficulty. The main effect of interval duration reported in this study is consistent with previous research in which the MMN elicited responses are dependent on the likelihood and degree of deviance of the stimulus to the standard (Kisley et al., 2004). Specifically, deviant stimuli that occur more infrequently and that are more distinct from the standard stimuli elicit the greatest responses. In addition, an interaction was detected, whereby the neurophysiological responses to the timed intervals were amplified by cognitive preparedness and directed attention to detect changes in interval durations in the attention conditions. The pattern of increased amplitude to a greater degree of deviance when paired with greater attention to the stimuli is similar to that of MMN responses to deviants in sensory modalities such as audition (Sussman et al., 2002). The current results provide further support for the idea that temporal processing is akin to basic sensory processing. Furthermore, the heightened electrophysiological responses of the MMN in the attended conditions suggest that attention or cognitive control can facilitate detection and processing of deviant temporal information.

The observed results suggest that endogenous types of timing tasks may receive a boost in neuronal response due to the task goals as the attended condition had increased responses particularly for the most deviant intervals. Based on previous findings in which MMN was elicited passively to temporal deviants, it is arguable that the increased response in the attended condition is most likely an effect of the task goals above and beyond the responses in the passive condition. Thus the observed difference between the attended and passive MMN responses suggests a difference between endogenous and exogenous implicit timing tasks. Moreover, the endogenous, attended condition allows for greater influence on and modulation of timed intervals, as evidenced by the interaction, than the exogenous, passive one. That is, neuronal responses to deviants that were less distinct than the standard were more readily detected in the endogenous tasks.

The current findings suggest that attention may modulate the MMN amplitude in terms or responses to temporal information, specifically when the task requires a greater degree of attentional resources than are required for a passive condition or a condition for which the overall goal is less demanding (low attention condition). It is hard to disentangle what role attention plays in the increased MMN amplitude, but results suggest that increased attention utilized as part of what might be considered an endogenous timing paradigm affected what has generally been viewed as an early brain response that can be elicited passively to changes in temporal information (Sussman et al., 2014). These findings are interesting in that the results suggest that when one is involved in a goal-directed paradigm or engaging in endogenous time processing, neuronal responses may be better prepared to track temporal and deviant information. It may be, as Sussman et al. (2014) describe, that the neural representations of stimuli in memory that are used in the MMN response are altered by task goals rather than how well one listens to the stimuli. But the current findings, along with additional studies of attention and/or task goals suggest that MMN may be affected by context to a greater degree than once thought. Specifically, while many studies of MMN in the past have supported what Cheour (2007) describe as the “automatic, bottom-up” nature of MMN, which orients attention toward stimuli and relies of the passive creation of the echoic trace and expectation, the current study provides evidence that MMN can be affected by “top down” processes (Cheour, 2007; Boutros et al., 2014).

The implications regarding the changes across components suggest that the neurophysiology of timing may be more malleable than once thought. Specifically, rather than interventions aimed at adapting to poor timing at the behavioral level, the current results suggest that improving timing skills should be addressed both at the behavioral level and at the neurophysiological level. It may be that individuals are simply more accurate when timing is taught or processed in the context of a goal. It may also be that the goal piece is secondary to the influence of the attentional load. What the results appear to support is that in addition to prior findings suggesting improvement in behavioral temporal accuracy via increased attention, that the neural underpinnings of time processing are also strengthened by attention.

Interestingly, the N2 and P3 results highlight differences in sensory and attentional aspects of the task paradigms, with the frontal N2 responding to stimulus driven novelty effects (Folstein and Van Petten, 2008) and the P3 responding to attentional and higher order demands (Polich, 2007). The MMN amplitude appeared to exhibit responses to both stimulus and attentional features. This supports the view that the MMN indexes both the establishment of a pattern and a violation, cued from new stimulus features, of that pattern. The MMN has been conceptualized as a component marking a shift in attention arising from novel or different sensory information (Näätänen et al., 2007). The current study reports the interaction at this sensory-attentional intersection. Further, the N2 exhibited a sensory influence and the P3 showed a marginal modification with attention. Thus, deficits observed in the MMN response may be able to be disentangled from stimulus features or attentional demands by tracking N2 and P3 in conjunction with the MMN. For example, it is possible that the development of the prediction model could falter in some clinical disorders whereas attentional influences may differentially influence the MMN in others.

The idea that different patterns of temporal performance or temporal dysfunction may affect different populations based on neural underpinnings is not a new idea. Wiener et al. (2010b) followed up on the work of Coull and Nobre by assessing brain function associated with implicit versus explicit temporal processing. Their research suggests that there are likely shared neural substrates associated with both types of temporal processing, but more important to the current study, there are also different patterns of brain activation elicited based on task features. While the current study only begins to inform us about the electrophysiological correlates of exogenous and endogenous temporal tasks, the findings a least suggest that greater investigation in to this topic is warranted. Additionally, one limitation of the current study is the exclusion of a greater range of duration deviants. While it was clear participants were engaged in the tasks, their behavioral performance suggested that the more difficult tasks may have been too difficult to achieve high rates of accuracy. Future studies including a wider range of difficulty in the behavioral tasks could distinguish levels of temporal information that is challenging both at a behavioral level and neuronal level. In addition, future work should manipulate task goals within populations who struggle with temporal information to assess if the endogenous/exogenous nature of the task may alleviate temporal processing problems.

### Conflict of Interest Statement

The authors declare that the research was conducted in the absence of any commercial or financial relationships that could be construed as a potential conflict of interest.
